# Assessment of cognitive function following a course of electroconvulsive therapy

**DOI:** 10.1192/j.eurpsy.2021.1306

**Published:** 2021-08-13

**Authors:** S. Ali, S. Kamel, J. Easow, R. Blasubramanian, P. Parvathy

**Affiliations:** Basildon Mental Health Unit Inpatients Grangewaters Ward, Essex partnership nhs trust, Basildon, United Kingdom

**Keywords:** ECT, MOCA, MADRS, Cognitive functions

## Abstract

**Introduction:**

ECT is a potentially life-saving treatment for patients with severe or treatment resistant depression. Cognitive function disturbances following ECT are generally transient, but could be of longer duration in some cases

**Objectives:**

To assess the cognitive side effects in patients with affective disorders treated with a course of electroconvulsive therapy (ECT).

**Methods:**

Cognitive functions of patients who undergo ECT was assessed prior to start of treatment, midway of the course of treatment and after end of the course of treatment using Montreal Cognitive Assessment (MoCA). We did a retrospective analysis of MoCA scores of 15 patients who received bilateral ECT in 2017-2018. In order to assess the efficacy of ECT in the treatment of their illness, we did a retrospective analysis of Montgomery Asberg Depression Rating Scale (MADRS) scores of 18 patients who received bilateral ECT in 2017-2018

**Results:**

Only 7% of the patients who underwent ECT in our sample did have significant cognitive decline as per their MoCA scores. 28% of patients achieved complete remission in their depressive symptomes. 22% of patients continued on maintenance treatment. 95% of patients showed significant improvement in their symptoms following treatment with ECT where there symptoms reduced to either mild or minimal depressive symptoms.

**Conclusions:**

Cognitive side effect was not a significant side effect in our sample of patients. We did see an improvement in cognitive function in a significant number of the sample of patients as they progressed with treatment, which coincided with improvement in their affective symptoms.

